# Supplementary material for: Crustal rheology controls on the Tibetan plateau formation during India-Asia convergence
Source: Nat Commun. 2017 Jul 19;8:15992. doi: 10.1038/ncomms15992 (PMC5524925; doi:10.1038/ncomms15992)
Supplement: Supplementary Information [file ncomms15992-s1.pdf]

Type of file: PDF
Size of file: 0 KB
Title of file for HTML: Supplementary Information
Description: Supplementary Figures, Supplementary Tables and Supplementary References

Type of file: PDF
Size of file: 0 KB
Title of file for HTML: Peer Review File
Description:

Supplementary Information

Supplementary Table 1 Conditions and results of 3-D numerical experiments for continental indentation

Model	V_x (cm yr^{-1})[1]	Lithospheric layering for indenter (km)[2]	Lithospheric layering for upper plate (km)[3]	Flow law for indenter[4]	Flow law for upper plate[4-6]	Comments
Model-1	3.3	UC: 15<br>LC: 20<br>ML: 105	UC: 17<br>LC: 23<br>ML: 81	UC: Wet Quartzite<br>LC: **Plagioclase An$_{75}$**<br>ML: Dry Olivine	UC: Wet Quartzite<br>LC: **Mafic Granulite**<br>ML: Dry Olivine	Wide plateau; used in Figs. 2, 4 and 5.
Model-2	3.3	UC: 15<br>LC: 20<br>ML: 105	UC: 17<br>LC: 23<br>ML: 81	UC: Wet Quartzite<br>LC: **Plagioclase An$_{75}$**<br>ML: Dry Olivine	UC: Wet Quartzite<br>LC: **Mryland Diabase**<br>ML: Dry Olivine	Narrow orogen; used in Figs. 2, 4 and 5.
Model-3	3.3	UC: 15<br>LC: 20<br>ML: 105	UC: 17<br>LC: 23<br>ML: 81	UC: Wet Quartzite<br>LC: **Plagioclase An$_{75}$**<br>**f**or $0<X<800$ km<br>ML: Dry Olivine	UC: Wet Quartzite<br>LC: **Maryland Diabase**<br>for $0<X<200$ km<br>LC: **Mafic Granulite**<br>for $200\leq X\leq 1000$ km<br>ML: Dry Olivine	Narrow orogen to the west and wide plateau to the east; used in Figs. 3, 4 and 5.
Model-4	2.0	UC: 15<br>LC: 20<br>ML: 105	UC: 17<br>LC: 23<br>ML: 81	UC: Wet Quartzite<br>LC: **Plagioclase An$_{75}$**<br>ML: Dry Olivine	UC: Wet Quartzite<br>LC: **Mafic Granulite**<br>ML: Dry Olivine	Wide plateau
Model-5	5.0	UC: 15<br>LC: 20<br>ML: 105	UC: 17<br>LC: 23<br>ML: 81	UC: Wet Quartzite<br>LC: **Plagioclase An$_{75}$**<br>ML: Dry Olivine	UC: Wet Quartzite<br>LC: **Mafic Granulite**<br>ML: Dry Olivine	Wide plateau
Model-6	2.0	UC: 15<br>LC: 20<br>ML: 105	UC: 17<br>LC: 23<br>ML: 81	UC: Wet Quartzite<br>LC: **Plagioclase An$_{75}$**<br>ML: Dry Olivine	UC: Wet Quartzite<br>LC: **Mryland Diabase**<br>ML: Dry Olivine	Narrow orogen
Model-7	5.0	UC: 15<br>LC: 20<br>ML: 105	UC: 17<br>LC: 23<br>ML: 81	UC: Wet Quartzite<br>LC: **Plagioclase An$_{75}$**<br>ML: Dry Olivine	UC: Wet Quartzite<br>LC: **Mryland Diabase**<br>ML: Dry Olivine	Narrow orogen

$_{\mathbf{x}}$, convergence rate; UC, upper crust; LC, lower crust; ML, mantle lithosphere. 5

Supplementary Table 2 Physical properties of rocks used in numerical experiments

Material	ρ_0[7,8], (kg/m^3)	Thermal conductivity[9] (W/m/K, at T_K)	$T_{solidus}$[8, 10-14] (K, at P_{MPa})	$T_{liquidus}$[8, 10-14] (K, at P_{MPa})	Flow law[4-6]
UCC	2700 (solid)<br>2400 (molten)	$0.64+807/(T+77)$	$889 + 17900/(P + 54) + 20200/(P + 54)^2$ at $P < 1200$ MPa,<br>$831 + 0.06P$ at $P > 1200$ MPa	1262 + 0.09P	Wet Quartzite, A_D=1.97×10^{17} Pa^{n}s, n=2.3,<br>E_a=154 kJ/mol, V_a=0 cm^3 mol^{-1},<br>C=1 MPa, sin(ϕ)=0.15
LCC for India	2800 (solid)<br>2500 (molten)	$1.18+474/(T+77)$	$1327.15+0.0906P$	1423 + 0.105P	Plagioclase An_{75}, A_D=4.80×10^{22} Pa^{n}s, n=3.2,<br>E_a=238 kJ/mol, V_a=0 cm^3 mol^{-1},<br>C=1 MPa, sin(ϕ)=0.15
LCC for Asia (weak)	2800 (solid)<br>2500 (molten)	$1.18+474/(T+77)$	$1327.15+0.0906P$	1423 + 0.105P	Mafic Granulite, A_D=1.58×10^{21} Pa^{n}s, n=3.2,<br>E_a=244 kJ/mol, V_a=0 cm^3 mol^{-1},<br>C=1 MPa, sin(ϕ)=0.15
LCC for Asia (srong)	2800 (solid)<br>2500 (molten)	$1.18+474/(T+77)$	$1327.15+0.0906P$	1423 + 0.105P	Maryland Diabase, A_D=1.98×10^{27} Pa^{n}s,<br>n=4.7, E_a=485 kJ/mol, V_a=0 cm^3 mol^{-1},<br>C=1 MPa, sin(ϕ)=0.15
Mantle	3300 (solid)<br>2900 (molten)	[0.73+1293/(T+77)]×<br>(1+0.00004P)	Melting model of Katz et al. (2003)	Melting model of Katz et al. (2003)	Dry Olivine, A_D=3.98×10^{16} Pa^{n}s, n=3.5,<br>E_a=532 kJ/mol, V_a=8 cm^3 mol^{-1},<br>C=1 MPa, sin(ϕ)=0.6

UCC, upper continental crust; LCC, lower continental crust; LAM, lithospheric/asthenopsheric mantle. Other properties (for all rock types): $C_p = 1000$ J kg^{-1} K^{-1}, $\rho = \rho_0[1 - \alpha(T - {}_0)] \times [1 + \beta(P - {}_0)]$, where $\alpha = 3 \times 10^{-5}$ K^{-1} is thermal expansion and $\beta = 1 \times 10^{-5}$ MPa^{-1} is compressibility; ρ_0 is density at room condition (${}_0 = 298$ K and ${}_0 = 0.1$ MPa).

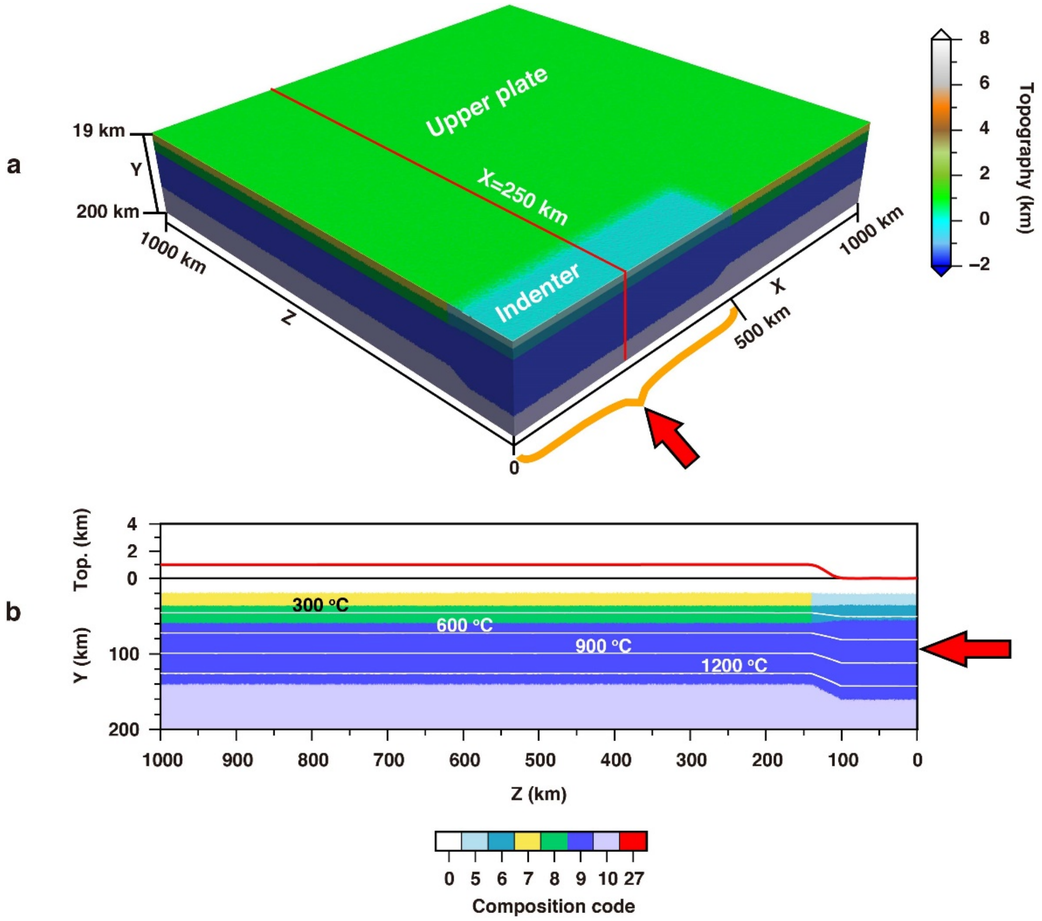

Supplementary Figure 1. Model setup and initial conditions. See Methods for details. **a**, 3-D model setup with the initial topography on the top, showing the configuration of the indenter (cyan) and upper plate (green). **b**, cross section through x=250 km, as denoted by the red line in **a**, showing initial composition and temperature distributions. Color codes for topography and composition are shown at the upper right side and bottom of the figure, respectively. For composition codes, 0-sticky air; 5-upper continental crust (indenter); 6-lower continental crust (indenter); 7-upper continental crust (upper plate); 8-lower continental crust (upper plate); 9-lithospheric mantle; 10-asthenosphere; 27-partially molten continental crust, which does not appear here, but may show during the model evolution. The red arrow shows the direction of convergence, and the orange parenthesis shows the region where convergence is imposed.

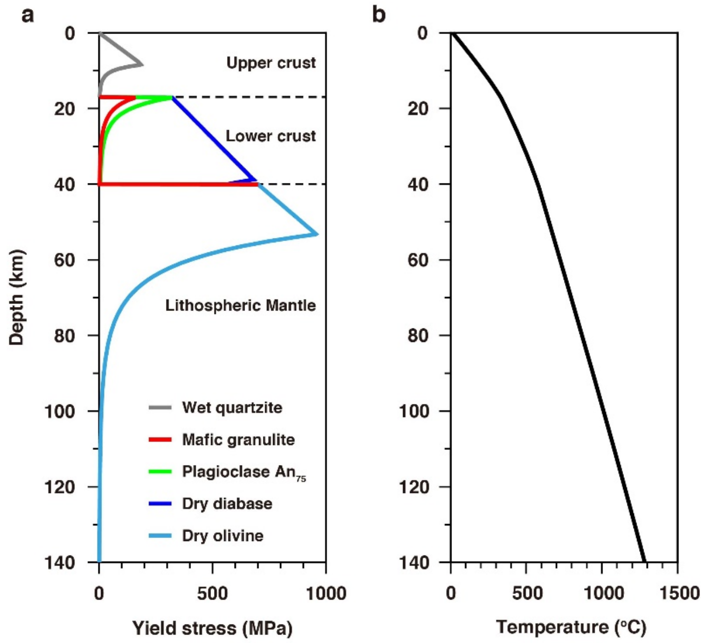

Supplementary Figure 2. Adopted flow laws for continental lithosphere. See Supplementary Table 2 for details. **a**, Yield stress envelopes showing end-member rheologies for the lower continental crust. 'Mafic Granulite', 'Plagioclase An_{75}' and 'dry Diabase' represent the weak, intermediate and strong crust, respectively. The upper crust and lithosphere mantle for all the experiments are represented by 'wet Quartzite' and 'dry Olivine', respectively. **b,** Typical geotherm for continental lithosphere, which is used to construct stress envelopes in **a**. The strength of the whole lithospheres is defined by the depth-integral of the stress profile, yielding values of 16.34 TN m^{-1}, for the Mafic Granulite, 17 TN m^{-1}, for the Plagioclase An_{75}, and 27.36 TN m^{-1} for the dry Diabase. The dry Diabase Asian crust introduces an increase of integrated strength of ~61%, with respect to the Indian lithosphere with Plagioclase crust, and is thus stronger. The Granulitic crust leads to a ~4% weaker Asian lithosphere with respect to the Indian lithosphere. The strength increase between Asian strong and weak lithospheres, with Diabase and Granulitic crusts, respectively, is 67%, showing that the rheology of the lower crust has a strong control on the whole lithospheric strength.

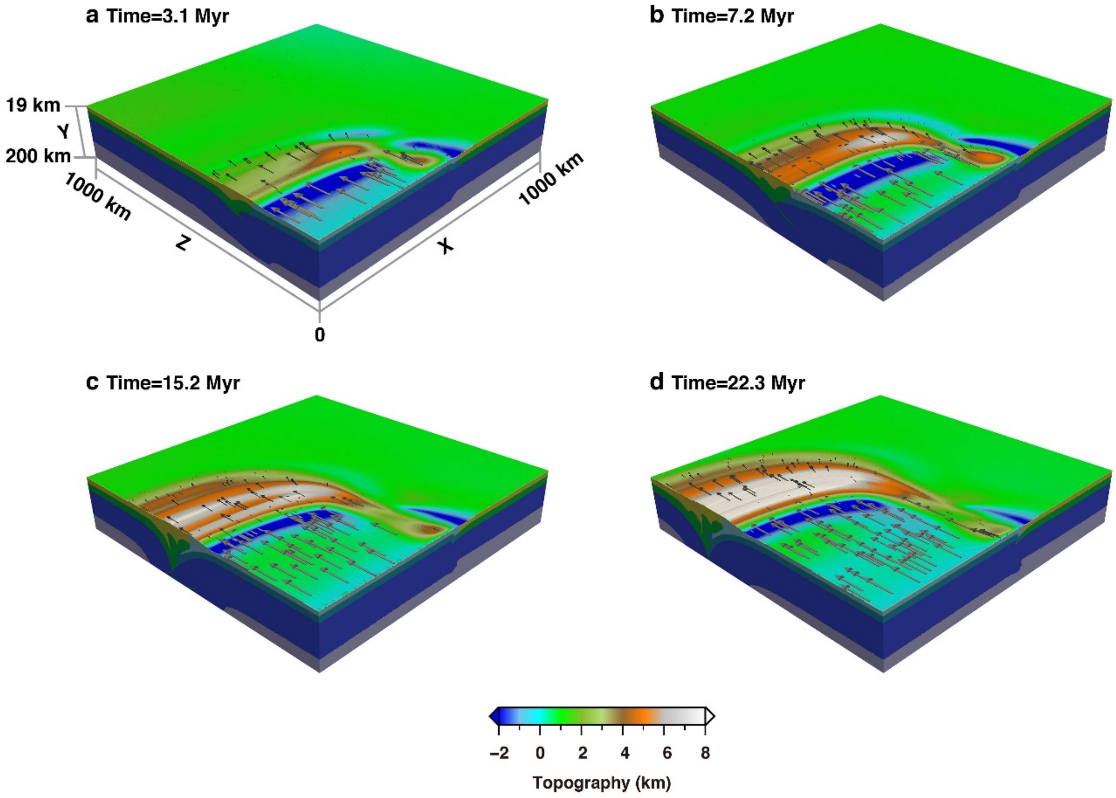

Supplementary Figure 3. Temporal evolution of the weaker crust model (Model-1). See Supplementary Table 1 for more details, and see Supplementary Figure 1 for composition code. The convergence rate here is 3.3 cm yr^{-1}.

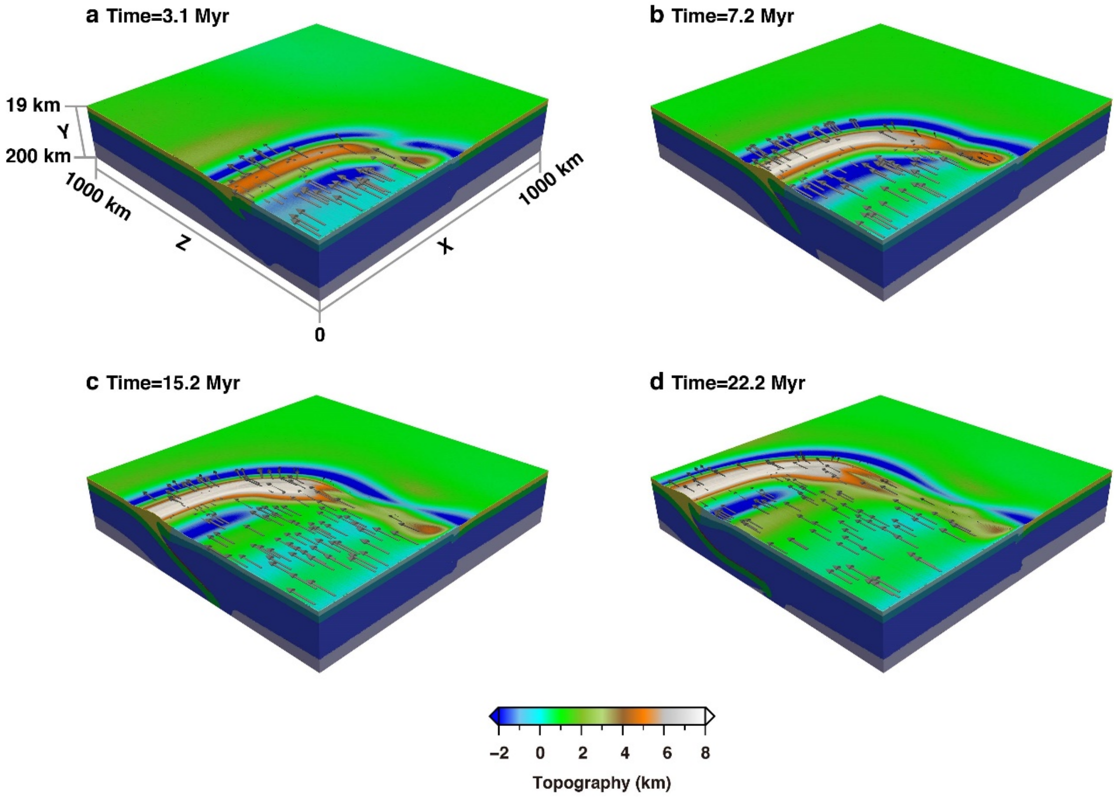

Supplementary Figure 4. Temporal evolution of the stiffer crust model (Model-2). See Supplementary Table 1 for more details, and see Supplementary Figure 1 for composition code. The convergence rate here is 3.3 cm yr^{-1}.

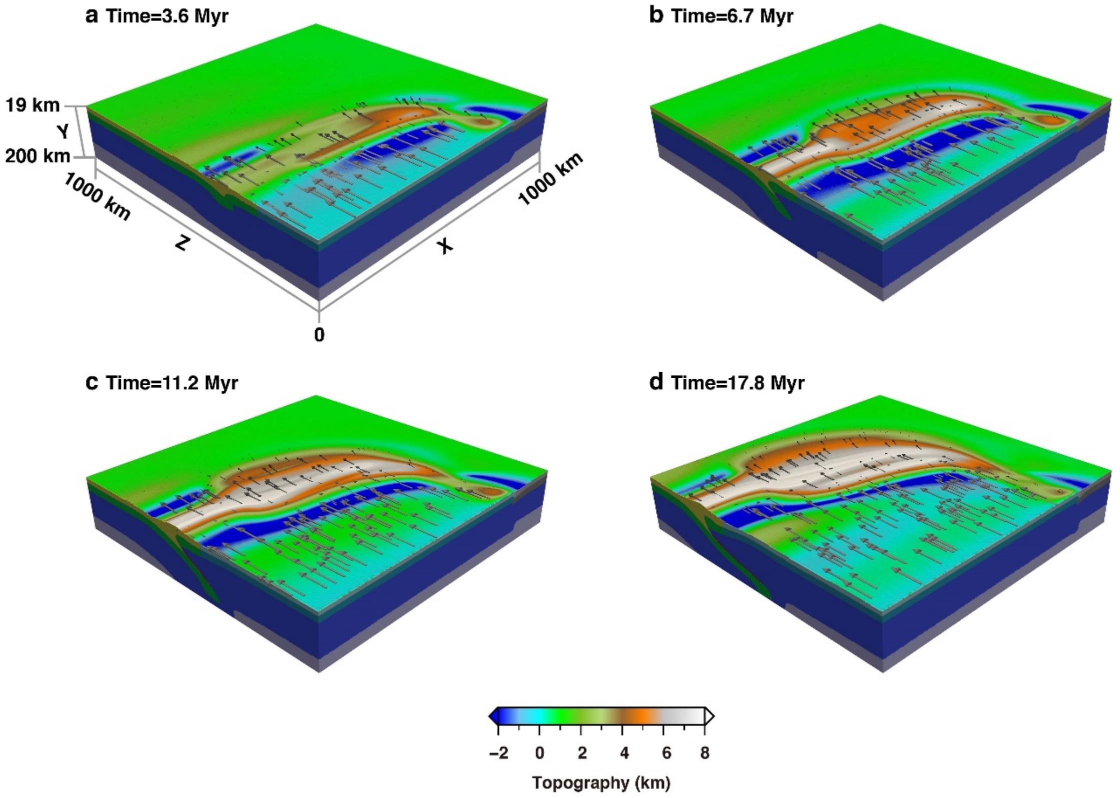

Supplementary Figure 5. Temporal evolution of the combined crust model (Model-3). See Supplementary Table 1 for more details, and see Supplementary Figure 1 for composition code. The convergence rate here is 3.3 cm yr^{-1}.

Supplementary References

1. Guillot, S. et al. Reconstructing the total shortening history of the NW Himalaya. Geochem. Geophys. Geosyst. **4** (2003).

2. Singh, A., Singh, C. & Kennett, B. L. N. A review of crust and upper mantle structure beneath the Indian subcontinent. Tectonophysics **644**, 1-21 (2015).

3. Hacker, B. R., Kelemen, P. B. & Behn, M. D. Continental lower crust. Annu. Rev. Earth Planet. Sci. **43**, 167-205 (2015).

4. Ranalli, G. Rheology of the Earth 2nd edn (Chapman & Hall, 1995).

5. Mackwell, S. J., Zimmerman, M. E. & Kohlstedt, D. L. High-temperature deformation of dry diabase with application to tectonics on Venus. J. Geophys. Res. **103**, 975-984 (1998).

6. Wang, Y. F., Zhang, J. F., Jin, Z. M. & Green II, H. W. Mafic granulite rheology: Implications for a weak continental lower crust. Earth Planet. Sci. Lett. **353-354**, 99-107 (2012).

7. Turcotte, D. L. & Schubert, G. Geodynamics (Cambridge Univ. Press, 2002).

8. Bittner, D. & Schmeling, H. Numerical modeling of melting processes and induced diapirism in the lower crust. Geophy. J. Int. **123**, 59-70 (1995).

9. Clauser, C. & Huenges, E. in Rock Physics & Phase Relations: A Handbook of Physical Constants (ed. Ahrens, T. J.) 105–126 (Am. Geophys. Union, 1995).

10. Johannes, W. in Migmatites (ed. Ashworth, J. R.) 36-85 (Blackie, 1985).

11. Schmidt, M. W. & Poli, S. Experimentally based water budgets for dehydrating slabs and consequences for arc magma generation. Earth Planet. Sci. Lett. **163**, 361–379 (1998).

12. Poli, S. & Schmidt, M. W. Petrology of subducted slabs. Annu. Rev. Earth Planet. Sci. **30**, 207-235 (2002).

13. Hess, P. C. Origin of Igneous Rocks (Harvard Univ. Press, 1989).

14. Katz, R. F., Spiegelman, M. & Langmuir, C. H. A new parameterization of hydrous mantle melting. Geochem. Geophys. Geosyst. **4**, 1073 (2003).
